# Evaluating spatiotemporal integration of shape cues

**DOI:** 10.1371/journal.pone.0224530

**Published:** 2020-05-20

**Authors:** Taylor Burchfield, Ernest Greene

**Affiliations:** Laboratory for Neurometric Research, Department of Psychology, University of Southern California, Los Angeles, California, United States of America; University of Wuerzburg, GERMANY

## Abstract

Prior work has shown that humans can successfully identify letters that are constructed with a sparse array of dots, wherein the dot pattern reflects the strokes that would normally be used to fashion a given letter. In the present work the dots were briefly displayed, one at a time in sequence, varying the spatial order in which they were shown. A forward sequence was spatially ordered as though one were passing a stroke across the dots to connect them. Experiments compared this baseline condition to the following three conditions: a) the dot sequence was spatially ordered, but in the reverse direction from how letter strokes might normally be written; b) the dots in each stroke of the letter were displayed in a random order; c) the sequence of displayed dots were chosen for display from any location in the letter. Significant differences were found between the baseline condition and all three of the comparison conditions, with letter recognition being far worse for the random conditions than for conditions that provided consistent spatial ordering of dot sequences. These findings show that spatial order is critical for integration of shape cues that have been sequentially displayed.

## Introduction

Experience dictates that humans can more readily perceive objects when the boundary cues are displayed in an orderly, systematic manner. It is likely that natural camouflage is effective because information about the object’s boundaries are spatially ambiguous and intermittent—as can occur when an animal moves behind vegetation [1]. The mechanisms providing for shape recognition often draw on the concept of closure proposed by Gestalt psychologists, wherein the parts of an object contribute to generating an integrated whole. When this integration is disrupted by inconsistent presentation of the shape cues, one is less likely to recognize that object, as edge detection is the first step in object recognition [2,3]

**Fig 1:**
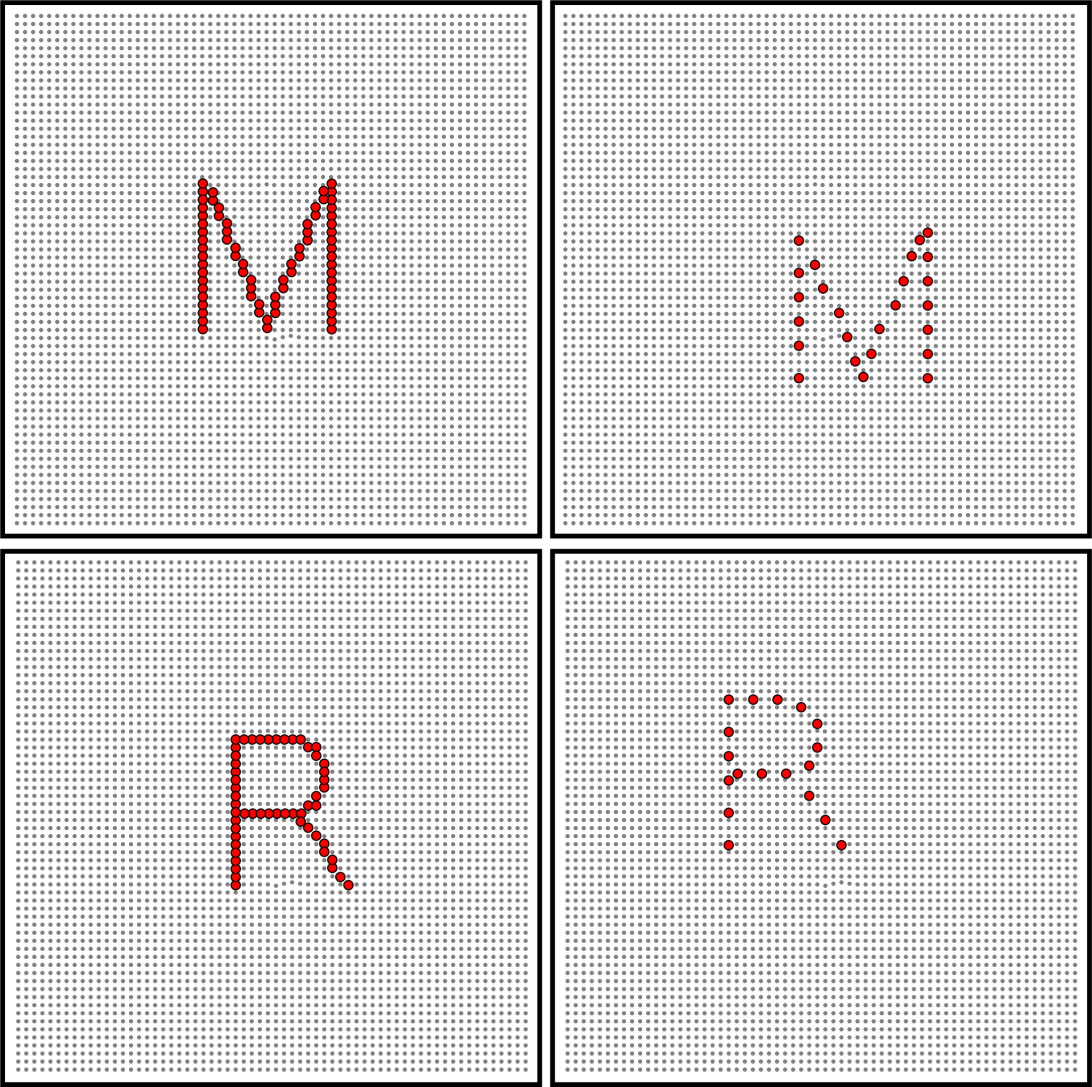
Illustration of dot density condition. The left panels show examples wherein the array of dots forming the letter strokes are at 100% density. The right panels show the low-density (30%) versions that were used in the experiment, wherein the dots were briefly displayed one at a time, with the order of display being varied.

Prior work has demonstrated that the human visual system is capable of perceiving shapes with minimal stimulus information, such as when the shape is represented by a pattern of dots and the display provides only a sparse “low-density” sample of the pattern [4, 5, 6]. The current study, and the previous studies to which it is related in this lab, used an LED board wherein the stimuli are presented via individual lights on that board. Therefore, in the construction of stimuli, multiple lights are used in order to create an image, much like pixels are used in digital media. A representation of this setup can be seen in Fig 1. Thus, in this case, “density” is meant to refer to the number of displayed dots in comparison to the number of dots required to fill the entire shape. For letters formed as single-file strings of dots, respondents manifest above chance recognition of the letters at 3% density. The performance is near-perfect at 27% density, i.e., wherein roughly every fourth dot in the string is included in the letter display [7]. This suggests that the information provided by the strokes normally used to create letters is highly redundant. The discrete strings of dots from which a low-density sample is drawn already has gaps between each dot, yet one can eliminate four out of five dots and still see near perfect recognition of the letters.

Humans have also been shown to be able to integrate shape information across an interval of at least 200 ms, with an ability to integrate stimuli across time starting in infancy [8]. This apparently innate human ability can be attributed to the phenomenon of information persistence, which is the availability of visible properties of the stimulus to the observer after the stimulus disappears [9,6]. This persistence allows the visual system to retain stimulus information for a sizeable temporal interval, allowing it to be integrated with related cues that are subsequently provided.

Given that the visual system can identify shapes from minimal cues and with temporal separation of those cues, the goal of this work is to more fully investigate how the sequencing of the cues affects the integration process. One might reasonably assume that systematic presentation of boundaries information would be essential to integration. [10] examined the role of ordered display of shape elements with a deletion/accretion paradigm, wherein a shape moves behind an occluding surface, sequentially modifying visibility of boundary cues. They confirmed that the human perceptual system contains mechanisms that compensate for fragmented boundary presentation, or spatio-temporal discontinuities. A subsequent study by [11] examined how the perception of an ambiguous moving stimulus can be revealed as it moves in a background with contrasting elements. Specifically, as time passes, white dots on a black background disappear and reappear in such a way that indicates that there is a black square moving through the space. Thus, through accretion and deletion of the white dots, one gets the impression that there is an object moving through the background, and can even determine the shape of that object though the background and object are the same color. This indicates that as time passes, the observer is required to maintain both visible and invisible stimuli in working memory in order to draw the conclusion that there is a black square moving in the space.

Here we use sequential displays of low dot-density letters to investigate the integration of stimulus information as a function of spatial positioning and temporal separation. Letters were used because they can be reliably created and identified from successive graphic strokes. Properly speaking, a “stroke” refers to a letter element that is continuous while being written

For example, an A can have three strokes, two that are diagonal and one that connects the diagonals near the center; a B consists of a vertical stroke and two curved strokes. It is worth noting that although letter-strokes can be written in different ways, our findings are based on averages taken across the full alphabet. It seems unlikely that variation in how a few of the letters might be written would make a substantial difference in the recognition differentials that we are reporting. The spaced dots of our displays were positioned such that a continuous stroke could be drawn to connect them. It is convenient to describe the spatial relationships as strokes, even though the dots were not connected. [See Methods for details on the operational definition of stroke-sequences used in the present work.]

The left panels of Fig 1 illustrates the strings of adjacent dots that would comprise a given letter at 100% density, and the right panels show examples at 30% density. Of each of the three experiments described below, a baseline condition was the sequential display of dots that followed the path that would produce a handwritten letter. The dots were briefly displayed one at a time, as though an unseen stroke was causing each to be briefly illuminated. Further, the order of successive strokes themselves matched how the letter could be graphically generated [See Methods] This baseline condition can also be described as the “forward” treatment condition, meaning that the display sequence followed the normal direction for writing each of the several successive strokes for a given letter.

Experiment 1 compared the baseline condition with a “reverse” condition, i.e., the dots were displayed in the opposite order with respect to the baseline condition. This reversed not only the order within a given stroke sequence, but the strokes themselves. Experiment 2 compared the baseline condition with a “random within strokes” condition. Here the order of strokes was the same as baseline, but the order of dot display within strokes was random. Experiment 3 compared the baseline condition with a “completely random” condition, where the order of successive dots was chosen at random from the low-density letter, irrespective of stroke membership. The experiments are numbered in the order of hypothesized recognition, with the reverse condition expected to have the highest hit rate, and the completely random condition hypothesized to have the lowest hit rate. Each condition had five different stimulus onset asynchronies (SOA) to provide a temporal handicap on recognition in order to evaluate the importance of spatial sequencing, The SOA ranged from 0 ms (wherein all of the dots were presented simultaneously) to 800 ms. Previous work from this laboratory found that recognition of shapes declines as a function of temporal separation of boundary markers [12].

## Results

Each of the experimental treatments was designed to produce a decline in recognition as a function of the amount of delay between successive dot displays. Mixed effect logistic regression confirmed a significant decline for each treatment at p < 0.0001 in each of the experiments. Stimulus onset asynchronies (SOA) (as a continuous measure), treatment condition and their interaction were considered as fixed effects and subjects were considered random effects in the mixed-effects regression models. We also tested for each model the quadratic and cubic components of the time interval, but we didn’t include their interaction terms with treatment condition to avoid overfitting. Only significant factors were kept in the final models. The linear, quadratic, and cubic components that were significant for a given treatment provide models of treatment effects in the following figures, along with 95% confidence bands.

**Fig 2:**
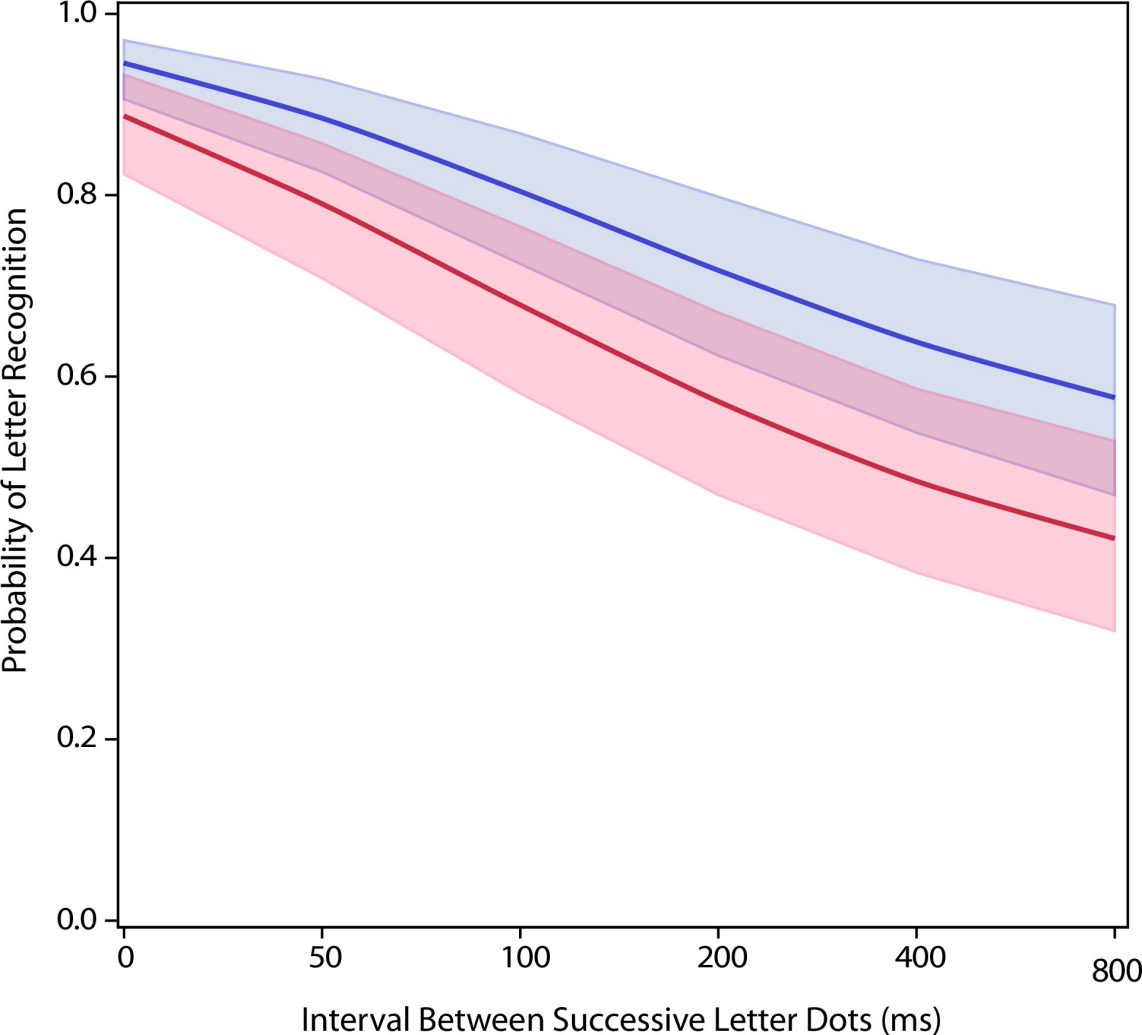
Exp 1 comparing a forward dot sequence to a backward dot sequence. Presenting the letter dots in a backward sequence (red) produced a larger decline in letter recognition than was produced by the forward sequence (blue)–p < 0.0001.

Experiment 1 evaluated how the interval between successive dots would affect letter recognition, comparing the effects of a forward sequence in relation to a backward sequence. The resulting models both show a dominant linear decline in recognition, with the rate of change being roughly the same for both treatments (p<0.0001 for linear decline; p = 0.51 for interaction between linear decline and experiment condition, see Fig 2 and Table 1 columns 2 and 3). Evaluating the treatment levels individually, the impairment was significant at 50 ms (overall effect size of the difference = 2.6, p<0.0001, see Table 2 Comparison #1) of dot separation and longer. The consistent differential in probability of recognition across all intervals greater than zero suggests that a forward sequence is better able to elicit memory of letter attributes. This may be related to the normal direction of eye scans in reading of written English, as will be discussed subsequently.

**Table 1:** Model fitting results for comparisons between dot sequence conditions.

	Experiment 1^†^		Experiment 2^‡^		Experiment 3^§^	
Model Parameters	F_1,2868_	P value	F_1,2867_	P value	F_1,2867_	P value
SOA	51.68	<0.0001*	144.52	<0.0001*	59.79	<0.0001*
SOA^2^	7.87	0.0051*	58.73	<0.0001*	12.05	0.0005*
SOA^3^	-		35.33	<0.0001*	4.56	0.0328*
Condition	17.92	<0.0001*	36.24	<0.0001*	101.05	<0.0001*
SOA x Condition	0.44	0.51	0.15	0.69	2.68	0.10

^†^ Comparing a backward dot sequence to a forward dot sequence.

^‡^ Comparing a random-within-stroke dot sequence to a forward dot sequence

^§^ Comparing a totally random dot sequence to a forward dot sequence

* significant difference, P value<0.05

**Table 2:** Overall effect sizes for comparisons between dot sequence conditions.

Comparisons		Overall Effect Size	t statistics	DF	P value
1	Experiment 1				
	Backward dot sequence vs. forward dot sequence.	2.6	7.39	2868	<0.0001*
2	Experiment 2				
	Random-within-stroke dot sequence vs. forward dot sequence	4.4	12.33	2867	<0.0001*
3	Experiment 3				
	Totally random dot sequence vs. forward dot sequence	7.9	22.40	2867	<0.0001*
4	Experimental conditions only				
	Backward dot sequence vs. random-within-strokes sequence	1.70	4.82	4291	<0.0001**
	Backward dot sequence vs. totally random sequence	1.87	5.28	4291	<0.0001**
	Random-within-strokes sequence vs. totally random sequence	0.16	0.47	4291	0.64^#^

* significant difference, unadjusted P value <0.05

** significant difference, adjusted P value <0.05

^#^ unadjusted P value

**Fig 3:**
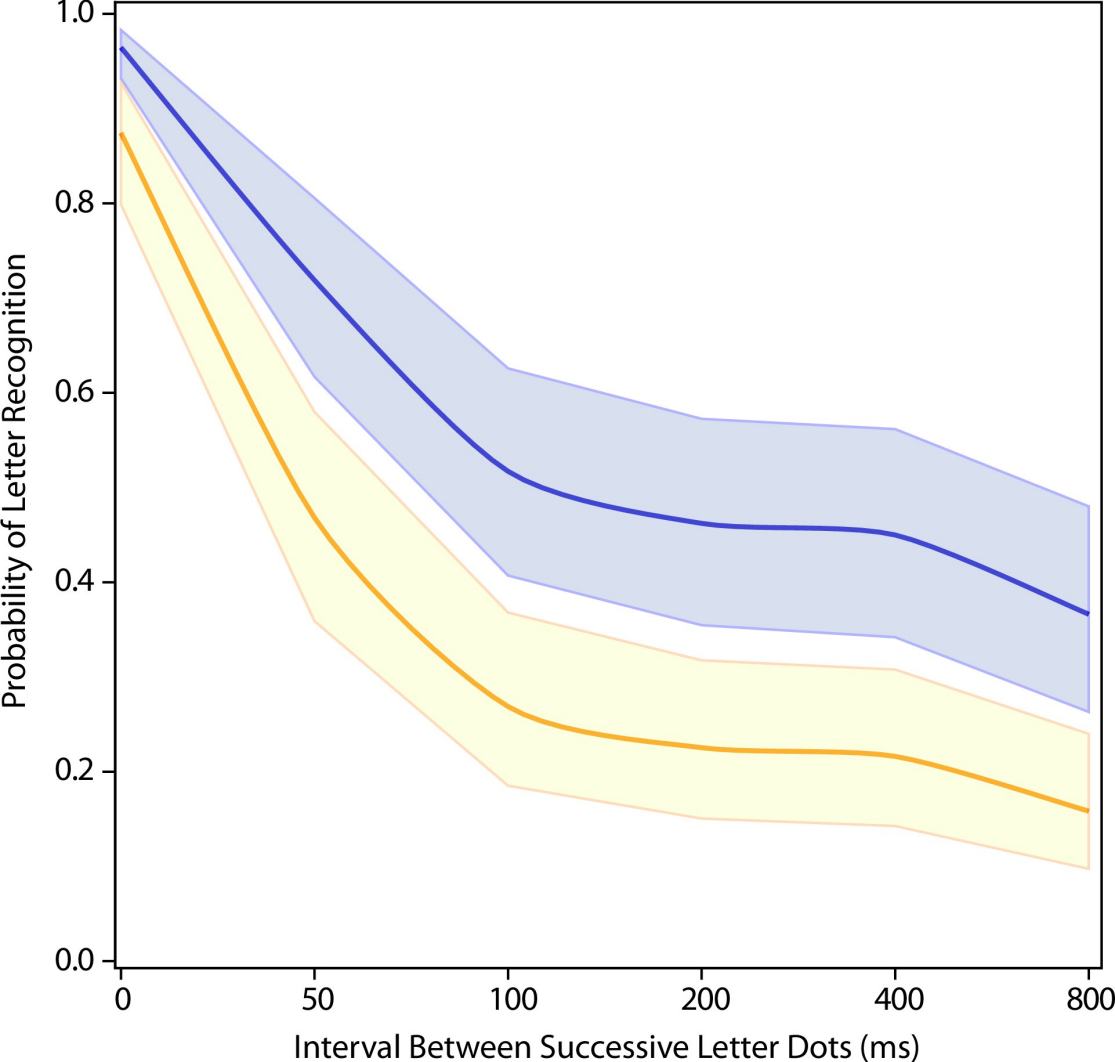
Exp 2: Comparing a random within-stroke dot sequence to a forward sequence. Presenting the letter dots in a random sequence within each stroke (gold) produced a larger decline in letter recognition than was produced by the forward sequence (blue)–p < 0.0001.

Experiment 2 compared the forward condition against a condition wherein the letter strokes were presented in the same order as the forward treatment condition, but with dots within each stroke being chosen in a random order. One respondent had mean letter recognition for the simultaneous (SOA = 0) display condition that was p < 0.0001 below the group mean, so the statistical analysis of treatment effect was done without including data from this respondent. The resulting model for the “random-within-stroke” condition also had large non-linear components, as can be seen in Fig 3 and Table 1 columns 4 and 5 (p<0.0001 for both linear, quadratic and cubic terms). Here also, the overall amount of impairment was well below that produced by forward sequencing of dots, starting at 50 ms (p<0.0001, see Table 2 Comparison #2)

**Fig 4:**
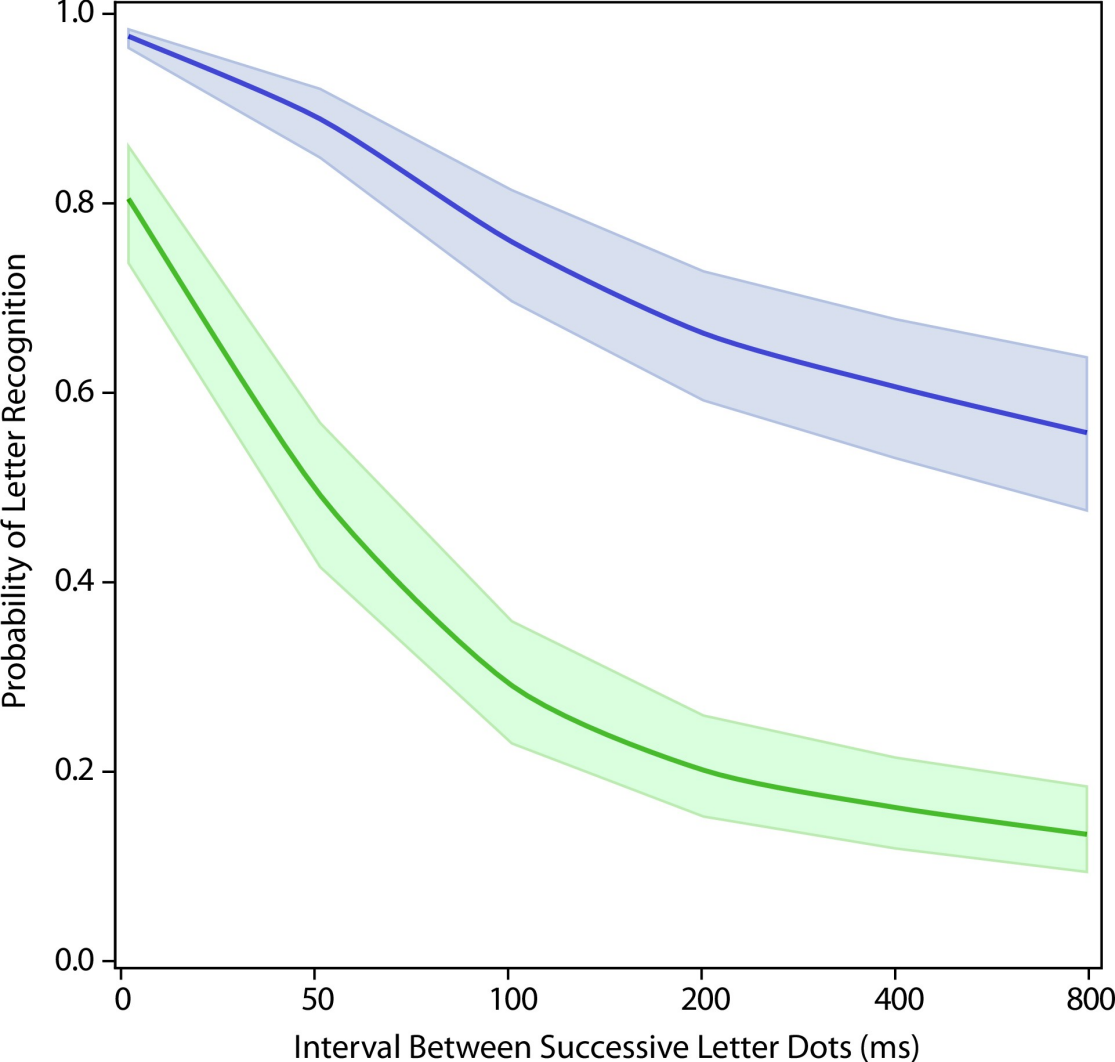
Exp 3: Comparing a random dot sequence to forward dot sequence. Presenting the letter dots in a random sequence (green) produced a larger decline in letter recognition than was produced by the forward sequence (blue)–p < 0.0001.

Experiment 3 evaluated letter recognition where the sequential dots were chosen at random from the full letter pattern, compared to presentation of dots using a forward sequence. One can see that the resulting model for the random condition adds a very strong quadratic component to the model, with recognition sinking to about 40% recognition with only 50 ms of dot separation (p = 0.0005 for quadratic component, see Fig 4 and Table 1 columns 6 and 7). Overall, the impairment of recognition for the random sequence can be seen to be substantially larger than was found in Experiment 1 starting at 50 ms (p<0.0001. see Table 2 Comparison #3). This differential is further analyzed below.

Presentation of dots as a forward sequence was the baseline treatment for each of the three experiments. The relative influence of non-baseline conditions was of special interest. The backward sequence (Exp 1, red) differed significantly from the random-within-strokes condition (Exp 2, gold) and the totally random sequence (Exp 3, green) starting at 50 ms (adjusted p<0.0001 between Exp1 and Exp2, and between Exp1 and Exp3, see Table 2 Comparison #4). However, the totally random sequence (Exp 3, green) did not significantly differ from the random-within-strokes condition (Exp 2, gold) (unadjusted p = 0.64, see Table 2 Comparison #4).

**Fig 5:**
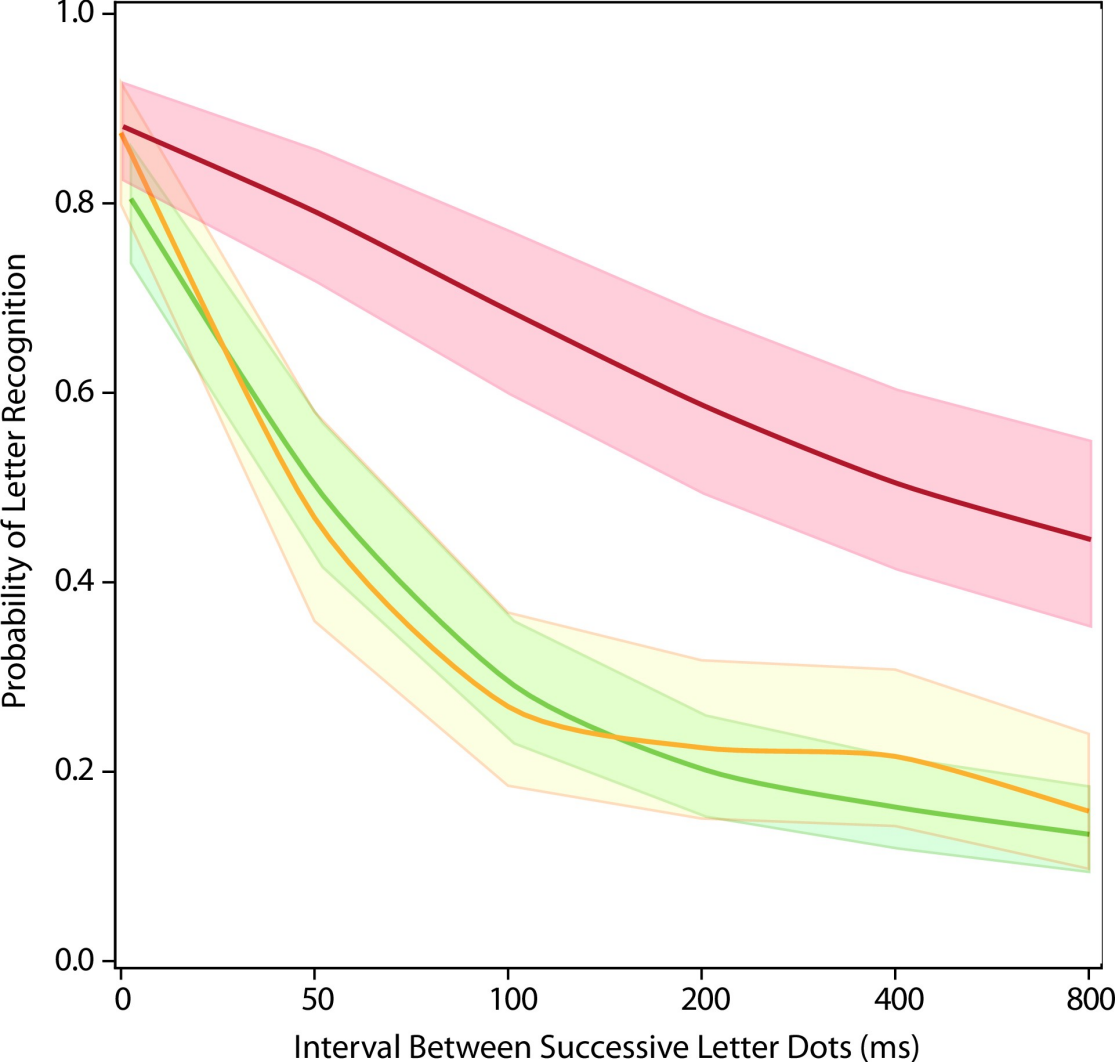
Comparing non-ordered sequences to the backward sequence. The presentation of successive dots as a backward sequence (Exp 1, red) differed significantly for the other two non-ordered conditions–p < 0.0001 for each comparison. The random-within-strokes condition (Exp 2, gold) did not differ significantly from the totally random sequencing of dot display (Exp 3, green).

Models showing the amount of recognition impairment for the latter comparison are illustrated in Fig 5. It seems clear that sequential display of dots selected from random locations greatly impairs letter recognition, and does so even if one has limited the choice to dots that all lie within a given stroke. The strokes that comprise a given letter are essentially its contours, and these results may well pertain to the broader topic of how the contours of diverse objects are registered for purposes of object recognition.

## Discussion

The three experiments demonstrated a significant difference between the baseline (forward) order of dot presentation and the other display orders that were evaluated. We therefore suggest that the forward sequencing of dots can be integrated more readily, allowing registration of the strokes on which the memory of letter-shape is based. This could be due to a lifetime of experience with writing letters as a forward sequence of strokes.

Further, we found that backward strokes yielded better recognition than either of the random conditions, which reflects an advantage for spatial ordering of the stroke markers. The ability to integrate the cues across time requires persistence of shape information, and the results show that visual system can better integrate a temporal sequence if the dots are adjacent. In other words, as successive dots are briefly flashed, their locations persist in the visual system for a short period of time. Display of adjacent locations within the dot pattern contributes to registering the stroke, and thus the letter. So, when the dots are presented in a consistent spatial order, the observer is able to better synthesize the stimuli, and can therefore perceive a more cohesive stimulus.

Visible persistence, which lies at the retinal level, can last for about a hundred milliseconds, whereas cortically based information persistence can provide for above-chance recognition for up to seven hundred milliseconds [6,13]. With these time periods in mind, we can speculate about the role of persistence in the integration of the present display sequences. At 30% density, the mean number of dots per stroke is just over six dots. A fifty-millisecond interval between successive dot would allow fourteen dots to last in working memory and a hundred millisecond interval would allow seven dots to be perceived. Therefore, most or all of the dots in a given stroke would be available to working memory. This could account for the minimal differential between forward and backward treatment conditions. However, it presents a challenge to the dramatically impaired performance for the random-within-stroke and random-within-shape conditions that were displayed with a temporal separation of only fifty milliseconds.

Information persistence failed to provide for recognition not only when the random selection of dots was from all portions of the letter, but also where the random choice was within a given stroke. The recognition deficits produced by the random display conditions can be attributed to “ineffective spatiotemporal integration,” in this case a failure to integrate brief displays of non-adjacent spatial information. These conditions prevent recognition of the letter shape even though all of the stimulus cues are simultaneously available to working memory. It is as though random presentation of the dots partially “cancels out” preceding and succeeding shape cues, which interferes with synthesis of the cues, and thus recognition.

[14] described this idea when investigating the phenomenon of illusory conjunctions. She found that objects having the same color, shape, or proximity to other similar objects yielded an illusory distinction between those items. As applied to the present displays, dots that were randomly ordered might be incorrectly paired, leading the observer to commit an error in processing the configuration. She related this phenomenon to the Gestalt principles of grouping, wherein the sum of parts would be expected to form a cohesive whole.

Another relevant process that could apply here is the accretion/deletion of shape cues. In nature, animate (moving) objects are often occluded by other elements of the scene, producing systematic, predictable sequences of boundary cues [15]. Presenting one dot at a time in sequence is similar to boundary cues being “deleted;” as an object moves behind an occluder [10] found that when objects moved through a complex scene with fragmented boundaries, recognition of the object declined as a function of the degree of fragmentation. In this study, they increased the number of target fragments, but not the number of noise fragments. They found that when there is a higher ratio of noise fragments relative to target fragments, recognition declined. They concluded that the extraneous elements from the scene were being incorporated into motion tracking mechanisms, which disrupted the accretion-deletion process. From this perspective, the ineffective spatiotemporal integration that our experimental conditions produced could be seen as an impairment of accretion/deletion mechanisms.

A subsequent study by [11] examined how accretion/deletion mechanisms could provide for the perception of moving shape, what is commonly described as “shape from motion.” For example, with a random pattern of white dots on a black background, one provides for the synchronized disappearance and reappearance of dots such that one perceives a black square moving across the field of dots. One sees the shape of the implied object even though it has the same brightness and color as the background.

The fact that humans show such sensitivity to this accretion-deletion phenomenon demonstrates that it is an integral element of motion detection and depth perception, starting from infancy. [16] discovered that infants use this principle in order to gauge depth at an edge, much related to work by [17] which investigated the reluctancy of infants to walk over a “visual cliff.” It is quite possible that the accretion-deletion mechanism, in this way, is crucial to survival in early life.

Related to accretion/deletion is perception of a scene through a moving slit. The ordered stroke condition most closely mimics the presentation of dots as seen through a moving slit—as the majority of the letter is concealed, the slit allows the observer to uncover shape elements in a systematic manner. [18] found that when a moving slit was presented over a line figure, participants are able to perceive a cohesive shape. Briefly presenting the stroke dots in a consistent spatial order emulates the temporary nature of seeing the shape boundary through a moving slit.

Even though the random-within-strokes condition presented strokes individually, it did not present the letters in a way that was “traceable;” i.e. it did not emulate how an object would appear and reappear in the environment, for it lacked continuity. Because of lack of perceived relation between the stimuli, this phenomenon resembles moving object tracking (MOT). A review by [19] included experiments which investigated MOT in the presence of indistinguishable decoy objects. They concluded that as the trial duration and the number of targets increased, subjects performed more poorly in identifying which of the objects was a target. In the present experiment, because the subjects had fewer cues connecting the dots in the random conditions, they might have treated these as multiple targets. In so doing, because the number of targets was considered to be increased in comparison to the forward and backward conditions, they had more difficulty tracking the dots.

Overall, the present results support the view that the visual system can encode sequential shape cues more effectively if they are spatially ordered. Considering that the visual mechanism for encoding shapes is likely the same as that for encoding letters, and that letters are formed using a fixed set of strokes, this study is a fitting candidate for evaluating the visual mechanism for encoding shapes as a whole. This is consistent with prior proposals that the initial stages of shape encoding are accomplished in the retina and/or superior colliculus, using scan waves to register the relative location of boundary markers [20,7,21,22,23,4]. This concept views elemental encoding of shapes as having evolved from primitive mechanisms for motion analysis, wherein successive portions of the shape’s boundary are registered as they pass across the retina. The process converts a two-dimensional shape into a one-dimensional signal that can be stored and used for subsequent recognition. Further evolution of this mechanism would yield the ability to generate scan waves that could produce the same kind of summary message in the presence of still images, i.e., where the shape to be recognized did not move. According to this view, effective recognition of a shape would require delivery of a spatially ordered sequence of shape cues, as was found here. Further relevant studies might compare the recognition rates between and within participants regarding the different letters. However, a potential issue with this study might include practice effects, which could influence responses.

## Methods

### Authorization, consent, and participation

The protocols for these experiments were approved by the USC Institutional Review Board. Respondents were recruited from the Psychology Subject Pool. Each respondent provided informed consent for being tested, which included provisions for termination of testing without penalty upon request by the respondent. A total of 24 respondents provided the data, eight in each of the three experiments.

### Display equipment

Letters were displayed as brief sequential flashes from a 64x64 array of LEDs (dots) mounted on a display board. Respondents viewed the display board at a distance of 3.5 m, and at this distance the visual angle of a given dot was 4.92 arc´, dot to dot spacing was 9.23 arc´, and the total span of the array (edge to edge) was 9.80 arc°. Ambient illumination of the room was 10 lux.

### Letter configurations

**Fig 6:**
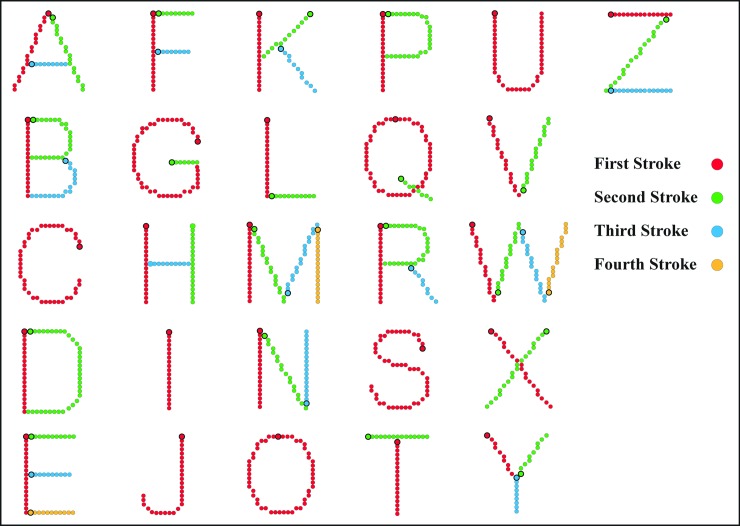
Alphabet showing the forward order of stroke sequences. The table of addresses represented letters of the alphabet as strings of adjacent dots (strokes), with most letters being constructed with several strokes. This illustration shows all the dots there were available in the address table for the letters, but the displays themselves provided a low-density sampling of the dots. For the baseline (forward) condition used in each of the three experiments, dots were presented in the order specified by the color code of this illustration. For a given stroke, the sequence began at the dot with a dark boundary. Using the letter Z as the example, the first stroke sequence (red) begins with the left dot; the second stroke sequence (green) begins at the top of the diagonal; the third stroke sequence (blue) begins with the left dot.

A table of dot locations represented letters as strings of adjacent dots, each letter being 20 dots tall and with a maximum width of 14 dots. It is convenient to describe the dot sequences as “strokes,” with the stroke sequences being configured as they might be written by hand. These stroke sequences are illustrated in Fig 6. For example, the letter A was specified as having three strokes, starting at the apex and proceeding down the left stroke, returning to the apex and proceeding down the right stroke, then starting at the middle of the left stroke and passing to the right to create the horizontal stroke. The letter B would be specified as beginning at the top dot to create the vertical stroke, then returning to the top to produce the upper loop-stroke, followed by the lower loop-stroke. The C was considered to be a single stroke, starting at the top-end dot and sequencing through each connected dot to end at the bottom end. Fig 6 illustrates the stroke specification for each letter of the alphabet.

In each of the three experiments, the letter pattern that was displayed on a given trial was a reduced-density (30%) sample of the full complement of dots available in the letter. Prior research [13] had found recognition to be asymptotic, near 100%, at higher dot densities. A 30% sample was expected to provide for 80–90% recognition when all the dots were displayed at the same moment, and the goal was to provide a decline from that high level as the time interval between successive dots was increased (see timing conditions, below). For each letter displayed, the 30% sample was chosen at random, on the fly, but with an algorithm designed to maximize spacing of adjacent dots within a given stroke. It is convenient to describe the 30% sample as a “stroke” even though it provides only some of the dots contained within the table of address locations.

### Letter display conditions

On each trial in each of the three experiments, positioning of a given letter on the display board was varied at random, with horizontal and vertical offsets of up to five dots. For a given display, the dots of the 30% sample were sequentially displayed, each as an ultra-brief flash at an intensity of 1000 μW/sr for a duration of 10 μs. The stimulus onset asynchrony (SOA) between successive dots was varied across six levels, these being: 0, 50, 100, 200, 400, and 800 milliseconds. A randomly selected letter was displayed at each of these treatment levels 30 times, for a total of 360 total trials. The experimental condition that distinguished the experiments was the order in which dots were selected for display, as follows.

Experiment 1 compared the “forward” condition with a “backward” condition. The forward condition ordered the dot sequence as illustrated in Fig 6, this being a baseline condition for each of the three experiments. For the “backward” condition the address list for the letter was read in reverse order, which not only reversed the sequence within each stroke but also the order in which strokes were delivered.

Experiment 2 compared the “forward” condition with a “random within stroke” condition, wherein the random choice was among the 30% sample of stroke dots, with the order of strokes being the same as for the forward condition.

Experiment 3 compared the “forward” condition to a “random” condition that displayed 30% of the dots from various dot locations within a given letter. The random selection of dot locations was done “on the fly” during testing, thus providing each subject with a different order of dot locations.

### Experimental test conditions

After receiving consent instructions, the respondent was seated against a wall of the test room, facing the display board, which was mounted on the opposite wall at eye level. A single pulse-width modulated light bulb was mounted about one meter above the respondent’s head, this providing ambient light for the room.

Each trial was preceded by a fixation marker, i.e., four dots at the center of the board emitting light at an intensity of 0.2 μW/sr. This marker disappeared immediately before display of the letter dots. The respondent was expected to say what letter had been displayed, and to guess if they did not recognize it or were unsure. In general this response was provided within 1–3 seconds, though the participants were not instructed to provide a response within a specific time limit. The test administrator then entered the letter that was named through a keyboard, which logged the response, along with specifics about which letter had been displayed and the treatment conditions for that trial. Entry of this information immediately launched the next trial, beginning with display of the fixation marker for half a second, followed by display of the next letter. The treatment conditions were randomized, such that after the forward condition, either the forward condition or the other condition could have been presented. Presentation of all trials generally took 40–45 minutes, and all respondents who were recruited for testing were able to complete all display trials.

### Availability of experimental data

Trial by trial decisions for each of the three experiments is available to the public on Open Source Framework under the project title “Spatiotemporal integration of shape cues,” deposited by Ernest Greene, January 31, 2020, https://osf.io/y4b29/
